# Validation of Total Mercury in Marine Sediment and Biological Samples, Using Cold Vapour Atomic Absorption Spectrometry

**DOI:** 10.3390/mps1030031

**Published:** 2018-08-23

**Authors:** Ahmed Abou Elezz, Hassan Mustafa Hassan, Hamood Abdulla Alsaadi, Ahmed Easa, Saeed Al-Meer, Khaled Elsaid, Zafar Khan Ghouri, Ahmed Abdala

**Affiliations:** 1 Environmental Science Center (ESC), Qatar University, P.O. Box 2713, Doha, Qatar; hassan.hassan@qu.edu.qa (H.M.H.); halsaadi@qu.edu.qa (H.A.A.); 2 Central Laboratory Unit, Qatar University, P.O. Box 2713, Doha, Qatar; ahmedaali@qu.edu.qa (A.E.); salmeer@qu.edu.qa (S.A.-M.); 3 Chemical Engineering Program, Texas A&M University at Qatar, P.O. Box 23874, Doha, Qatar; Khaled.elsaid@qatar.tamu.edu

**Keywords:** total mercury, validation method, IUPAC, absorption spectrometry, quantification

## Abstract

A method for the measurement of total mercury (T-Hg) in environmental samples using cold vapour atomic absorption spectrometry (CV AAS) has been validated yielding a dynamic range (0.04–10.00 μg/kg) and high certified reference material (CRM) recovery (>90%). The validation was carried out according to International Union of Pure and Applied Chemistry (IUPAC) validation and Eurachem Guides. A freeze-dried and homogenised sample was weighed and then digested using Suprapur acids (HNO_3_, H_2_SO_4_, and HF) with potassium dichromate solution in a hot block digestion system. A calibration curve was constructed (R^2^ > 0.999). Two CRMs (Marine Sediment Reference Material (PACS-3) and Trace Elements in Muscle Tissue (Trace Elements and Methylmercury in Mussel Tissue (NIST2976)) were utilised for quality assurance and control. The limit of quantification (LOQ) calculated as 0.04 µg/kg, and uncertainty (U) calculated as 2%. The obtained results showed the suitability of this method for direct mercury measurement in environmental samples. Additionally, the proficiency of this method was recognised by accreditation under the standard of International Organization for Standardization (ISO/IEC 17025:2017) for competence of testing and calibration laboratories.

## 1. Introduction

Mercury is recognised as one of the most hazardous environmental pollutants. It is released into the environment by anthropogenic and natural sources such as: volcanoes; industrial runoffs from contaminated soils; as well as gold and ore mining. According to the U.S. Environmental Protection Agency (US EPA), concentrations of total mercury (T-Hg) in biological samples are usually less than 0.1 mg/kg [1], while levels in sediment vary depending on the state of pollution of the area under study, the proper assessment of which necessitates the analysis of a large number of samples. Thus, there is a need for a robust, validated and inexpensive analytical method. Moreover, these methods should match the levels of total Hg that are hopefully, expected to be low.

The most frequently used protocols to determine total Hg in some biological samples employ atomic absorption spectrometry [2] and inductively coupled plasma mass spectrometry (ICP-MS) [3]. However, these methods require an additional sample preparation step that, significantly increases the analysis time and may be a source of contamination. Furthermore, there are some issues encountered when ICP-MS is used in the determination of total Hg. The use of the mercury analyser instrument model-AULA-254 is one of the recent alternatives to perform the T-Hg determination. Its uses include the evaluation of various environmental samples such as water, soils, sludge, and the analysis of foodstuffs and biota.

The analysis principle is based on a continuous flow method, where the mercury within the sample is converted into its elemental state with the aid of a reducing agent. The mercury is then stripped and carried to an optical cell, where the determination is performed by UV absorption at a wavelength of 253.7 nm. This technique is more commonly known as cold vapour atomic absorption spectrometry (CV AAS) [4].

In general, sensitive analytical methods are required for validation of traces of mercury in sediment and biota, since its concentration may be too low to be analysed by conventional methods.

The study aimed to develop and validate a robust method used to prepare and analyse various environmental samples. The novelty of the proposed method lies in its capability to estimate T-Hg content in a variety of environmental samples (water, sediment, and biota) following set sample preparation steps.

The quality of the proposed method has been checked by performing a complete method validation using set International Union of Pure and Applied Chemistry (IUPAC) criteria and employing two CRMs, its application to real samples has been illustrated by processing several environmental samples from the marine environment.

## 2. Material and Methods

### 2.1. Apparatus, Chemicals, and Reagents

Cold vapour atomic absorption spectrometry was utilised for T-Hg measurement, using an AULA-254 Gold (Mercury instruments GmbH Analytical Technologies, Karlsfeld, Germany), equipped with an electrodeless low-pressure mercury UV-absorption source set at a wavelength of 253.7 nm and running AULA-254 software (AULA-WIN, TM based) [5] for data processing. Argon 99.999% (Buzwair Scientific and Technical Gases, Doha, Qatar) and high-quality type 2 deionised water (~18 MΩ cm) from Thermos Barnstead system (GENPURE UV-TOC Life Technologies Ltd. Paisley, UK) were used. Other reagents such as Suprapur nitric acid (65%), sulfuric acid (98%), hydrofluoric acid (40%), an oxidising reagent (Potassium dichromate, EMSURE^®^ ACS), potassium permanganate (EMSURE^®^ ACS, Reag), hydroxylamine hydrochloride (99.999% trace metals basis), and tin-II-chloride (≥99.99% trace metals basis) were all obtained from Merck (Boston, MA, USA). Certified reference material (CRM) NIST-2976 (mussel tissue (trace elements and methylmercury), certified value: 0.061 ± 0.0036 mg/kg) were sourced from the National Institute of Standards and Technology (Gaithersburg, MD, USA). CRM PACS-3 (Marine Sediment Reference Material for Trace Metals and other Constituents), certified value: 2.98 ± 0.36 (mg/kg) was purchased from the National Research Council Canada (Ottawa, ON, Canada). Mercuric nitrate standard was used as a stock calibration standard (1001 ± 2 pg/mL, 2%, nitric acid in low TOC water (<50 ppb)).

### 2.2. Sample Preparation

The Environmental Science Centre has adapted the following procedure in preparing samples for T-Hg analysis. Approximately 0.1 ± 0.05 g of freeze-dried and homogenised sample was transferred into a 50 mL Teflon tube, 1 mL HF was added together with 5 mL HNO_3_, and 3 mL H_2_SO_4_ (Suprapur, Merck) to decompose and release matrix-bound mercury. The sample was then digested using a hot block set at 125 °C for 12 h with the cap closed to reduce mercury loss by volatilisation. After complete digestion, indicated by a clear solution, the tube was left to cool to room temperature after which 1 mL of resulting mixture was transferred into a 50 mL measuring flask together with 2 mL potassium dichromate, and the final volume made up to the mark with reagent water. This constitutes the sample solution. Other protocols employ different sample preparation techniques that may require different sample weights, utilise different digestion acids and other digestion apparatus [6,7].

### 2.3. Sample Analysis

Ten mL of the prepared sample was introduced into the AULA-254 for analysis. The process is automated in which the sample is oxidised, heated, reduced and finally analysed for T-Hg.

Moisture content determination of the environmental sample matrices was also carried out by a drying procedure (105 ± 2 °C for 48 h) using a validated and ventilated oven. Ten samples were used to calculate the mean moisture content with minimum sample mass 1 g [8,9].

## 3. Results and Discussion

### 3.1. Method Validation

The method was validated according to the IUPAC guideline, and Eurachem Guides, which include characterisation of selectivity, trueness, recovery, precision (repeatability & intermediate precision), limit of detection, limit of quantitation, linearity, range, and ruggedness [10,11]. The uncertainty of measurement was also calculated. The validation process was performed by analysing two different CRM matrices (PACS-3 and NIST-2976).

#### 3.1.1. Selectivity

Selectivity is the degree to which a method can quantitatively select a distinct analyte in the presence of other analytes that may interfere with the analyte under investigation. Matrix effect was investigated by analyzing two CRMs; each has a different matrix (muscle tissue and marine sediment). Interferences can result from anions or matrix. The main interference anion is chloride and sulfide will be absorbed in the same wavelength of mercury causing a positive interference. The matrix effects can be of two types; mask effects or background effects. Chloride and sulfide anions can be eliminated by adding potassium permanganate [12,13]. 

**Figure 1:**
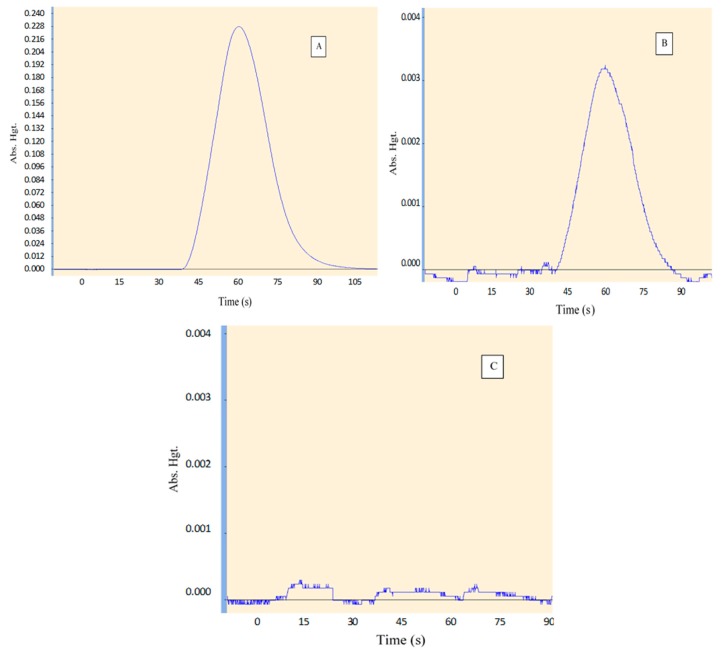
Spectrum of total mercury (T-Hg) analysis in two matrix reference materials at two different levels (high (**A**), low (**B**)) and blank sample noise background (**C**).

Two distinct peaks were observed from the CRMs previously described in Section 3.1, for low and high levels of T-Hg with no significant interference from other constituents within the CRMs with recovery > 90%. Figure 1 shows a typical of mercury at high (A) and low (B) levels (7 and 0.01 µg/L respectively) and a blank (C) with low noise and low background (absorbance < 0.001). The only T-Hg peak appears at the same retention time (~60 s) in the, from two prepared CRM samples containing other constituents.

#### 3.1.2. Trueness

Trueness relates to the systematic error of a measurement system and to how the mean value of replicate measurements are close to the true value. Eight measurements from two CRMs were analysed for bias; Table 1 shows the results of the trueness test on different days. The data obtained shows a relative bias of 4%, and −9% for NIST2976 and PACS3 respectively (Table 2).

The relative bias was calculated from Equation (1). Zeta score Equation (2) considers the reference material and the lab uncertainty. Table 2 shows that the relative bias in both CRMs (9).

(1) $$\mathrm{Bias}\%=\frac{\left(X_{lab}-X_{ref}\right)}{X_{ref}}\times 100$$

(2) $$Z=\frac{\left(X_{lab}-X_{ref}\right)}{\sqrt{((}SD{)}^{2}+\left(u_{ref}{)}^{2}\right)}$$

where:

*X_lab_* = Mean of Measurement

*X_ref_* = Mean of certified value

*SD* = Standard deviation of replicates

*u_ref_* = Standard certified uncertainty

#### 3.1.3. Recovery and Matrix Effects

Determination of T-Hg in environmental samples directly is problematic due to a matrix effect. The determination of mercury in these matrices (especially sediments and soil) accurately often requires decomposition of the matrix and conversion of all mercury forms to Hg(II), which can be reduced quantitatively to Hg^0^ [14].

High and low levels of concentration and different matrices were used for recovery calculations. Table 3 provides a comparison between our proposed method and traditional methods [7,15] of total mercury analysis by using CV-AAS and comparable matrices. Our method represents a high average % recovery (>95%) in both CRM matrices and relative standard deviation (RSD) less than or equal 5%.

#### 3.1.4. Precision

The precision character of measurement includes repeatability and intermediate precision. The RSD of replicates was used to evaluate the mean concentration of independent samples, and it shall not exceed 10% [16,17], and its calculated as (Equation (3)):

(3) $$\mathrm{RSD}=\frac{\left(Std\right)}{Mean}\times 100$$

##### Repeatability

Random error calculated from five repeated spiked blanks at three different levels (0.2, 2, 8 µg/L) through one day of analysis was used to assess the repeatability. Table 4 shows the RSD obtained from all CRMs replicates less than 10%, and full fill the criteria [16].

##### Intermediate Precision

Random error converts to systematic error over an extended period [17]. Intermediate precision is used to represent this type of error produced as a result of different operators, lab conditions, and batches of chemical reagents for an extended period. Table 5 shows the RSD obtained from eight CRMs (PACS-3 and NIST2976) analysis results performed over a periodic interval was less than 10% and full fill the criteria [17].

Generally, RSD_Repeatability_ is less than or equal to RSD_Intermediate precision_ [17].

#### 3.1.5. Limit of Detection and Limit of Quantitation

The detection limit is defined as “the lowest concentration of an analyte in the test sample that can be reliably distinguished from zero”. While the LOQ, defined as “the concentration below which the analytical method cannot operate with acceptable precision”. Low-level mercury spiked blanks concentration (0.01 µg/kg) were used to calculate the limit of detection (LOD), and limit of quantitation (LOQ) form a series of ten replicate [11,16]. The LOD was calculated as 0.01 µg/kg three times the standard deviation of the replicates and LOQ calculated as 0.043 µg/kg as ten times the standard deviation of the same replicates [16].

The mean concentrations of the spiked blanks were 0.014 µg/kg, and the signal to noise ratio (*S*/*N*) was equal to 3.16 (Equation (4)):

(4) $$S/N=\frac{\bar{x}}{\delta }$$

where:

$\bar{x}$ = the mean of replicates.

δ = The standard deviation of the replicates.

#### 3.1.6. Linearity and Working Range

Linearity was evaluated in a range of 0–150% of the analyte concentration. In this range, six evenly spaced calibration standards should be used. Each standard was analysed five times, and measurements carried out in random order.

**Figure 2:**
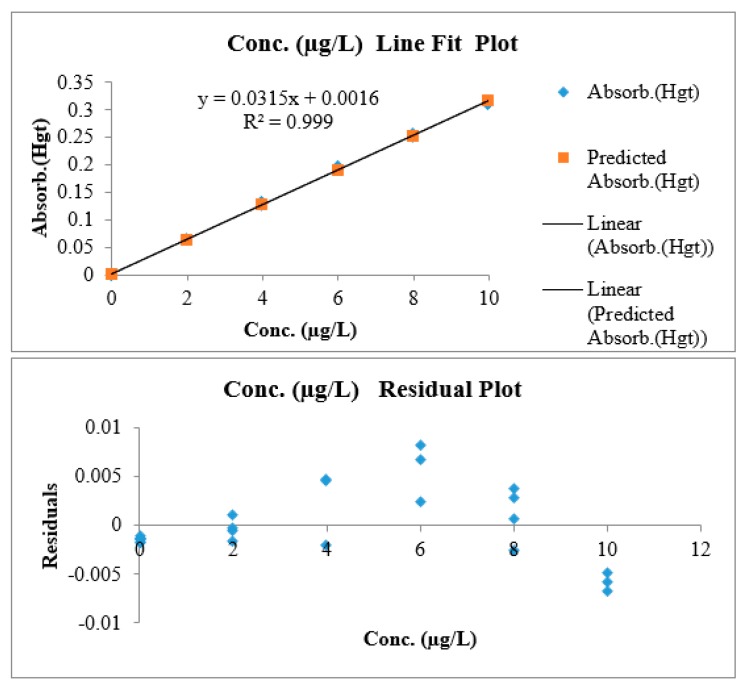
Calibration and residual charts for linearity lack of fit-test.

Linearity is evaluated based on visual inspection plot of residuals, random distribution of residuals about the zero confirm linearity. (Figure 2). The second way for evaluation was *F*-test (lack-of-fit) [11,18].

Table 6 shows seven independent calibration curves each point carried out five times. The working range was between 0.043 μg/kg–10 μg/kg with an R-squared equal to 0.9997.

The *F_Cal_*-equation was used to ascertain the linearity; If the (*F_Cal_*) is higher than (*F_Tab_*), the model cannot be considered fit for the data. i.e.,

H_0_ there is no lack of fit (linear).

H_A_ there is a lack of fit (nonlinear). Tabulated *F* value (*F_Tab_*) and calculated *F* value (*F_Cal_*) were calculated as per Equation (5). The *F_Cal_* value was 0.42 and *F_Tab_* was 2.56. Lack of fit is not statistically significant, and H_0_ hypothesis was accepted.

(5) $$F_{Cal}=\frac{MSS_{Lof}}{MSS_{error}}$$

where:

$MSS_{Lof}$ = Mean sum of squares due to lack of fit.

$MSS_{error}$ = The Mean sum of squares of random error.

#### 3.1.7. Ruggedness

The ruggedness of the method was examined by changing the principal factors, which have a direct influence on the analysis results such as temperature, digestion time, dilution, sample weight, reagent types, and amounts. Six experiments were performed for two different sample matrices and the Plackett–Burman [19] factorial design analysis for method ruggedness applied to evaluate the effect of changing the five factors (temperature, time, dilution, sample weight, and the amount of reagent added) on the analyte (T-Hg) result stability (Table 7 and Table 8). 

**Figure 3:**
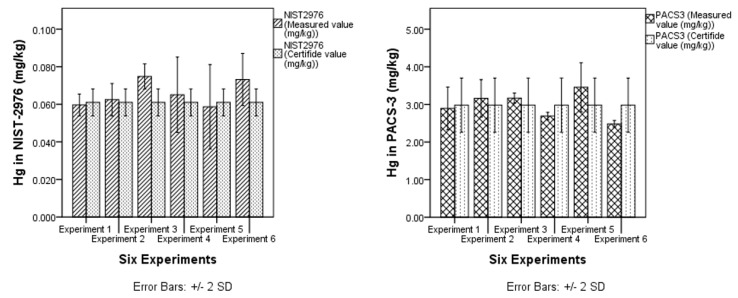
Mean concentrations of two different CRMs (NIST2976, PACS3) compared with its certified values in six experiments at different preparation conditions.

Each factor was tested at two levels, high (+) and low (−). Figure 3 shows a comparison between the measured and certified concentration values (mg/kg) from the two CRMs at different experimental conditions while Table 9 and Table 10 represent the statistical analysis (using analysis of variance (ANOVA)) of the two measured and certified mean values.

## 4. Estimation of the Measurement Uncertainty (Quantifying Uncertainty in Analytical Measurement)

Relative uncertainty is expressed as a relative standard deviation [20] and is equal to absolute error divided by the measured value. The quality of an analytical result is governed by measurement uncertainty, which defines an interval associated with the measured value, defining where this value lies with some probability. Similarly, expanded uncertainty is considered for Type-A and Type-B evaluation of uncertainty to estimate the main resources of error affecting the analysis [20]. Type-A based on statistical analysis of standard deviation of the mean replicates ($S_{\bar{x}}$) Equation (6).

(6) $$S_{\bar{x}}=\frac{s}{\sqrt{n}}$$

It covers CRM calibration standard repeatability uncertainty. Type-B evaluates all other non-statistical analysis, and it covers glassware uncertainty, equipment uncertainty, reagents, CRM and laboratory conditions uncertainties. Table 11 represents Type-A and Type-B uncertainties at confidence interval of 95% (*k* = 2). The relative standard uncertainties were divided by the divisor (probability distribution factor) before calculating the combined uncertainty. Combined uncertainty (u) was calculated as a sum squared of standard uncertainties from Equation (7). The relative expanded uncertainty (U) was calculated as 2% by using Equation (8) at 95% confidence interval (*k* = 2) (Table 11).

(7) $$u_{c}=\sqrt{\sum u_{R}^{2}}$$

where: *u_R_*= Relative standard uncertainty.

(8) U=(k×u)×100

## 5. Validation Characteristics Evaluation

Laboratories must ensure that all data they provide is of recognised quality. One aspect is the use of method validation and its evaluation. The typical steps for of a new test method validation include selectivity, trueness, precision, recovery, etc. The section below details the performed validation criteria (Table 12). Selectivity is the degree to which a method can quantitatively select a distinct analyte in the presence of other analytes that may interfere with our spectrum; two distinct peaks were observed from the CRMs, for low and high levels of T-Hg with no interference from other constituents within the CRMs.

Trueness assesses the closeness of the results obtained from the certified value.

Within our analysed results, the data obtained a relative bias of (4–9%), with acceptance criteria of ±10%. The data was also subjected to a zeta score analysis with results of (0.4–1.0) with acceptance criteria of not more than 2. The recovery acceptance criteria indicating a ±10% of the reference value [21] has also been met. Our result recovery ranged from 96–96.4%.

Precision, which included both repeatability and intermediate precision, was evaluated using RSD from five replicates at three different levels (0.2, 2, 8 µg/L), with results of 5, 2 and 1% for repeatability. Intermediate precision was evaluated using RSD from nine CRM (PACS3 & NIST2976) replicates producing 5.44 and 4.80%. Limit of detection and quantitation was calculated using multiples of standard deviation [16,22]. Our results produced LOD of 0.010 µg/kg, while the LOQ was calculated as 0.043 µg/kg. Signal to noise ratio for spiked samples should occur typically between 2.5 to 10 [16,23], our *S*/*N* was calculated as 3.16.

Linearity was assessed using lack-of-fit test [18]; employing a mean of the sum of squares [22] and an *F*-test ((*F_Cal_*) and (*F_Tab_*)) to examine the criteria acceptance.

As the obtained (*F_Cal_* = 0.42) is less than (*F_Tab_* = 2.56), we accept the null hypothesis and the calibration curve was linear.

Ruggedness was assessed by changing some essential factors that have a bearing on the analysis: such as temperature, digestion time, dilution volume, sample weight and reagents volumes, and employing the Plackett–Burman test and an ANOVA to evaluate the extent to which these variations affect the method. The results from the one-way ANOVA test showed that there are no significant variations between six experimental method groups for the two types of CRMs (*p* > 0.05) at confidence interval 95% illustrating that this method is robust under varying conditions.

Measurement uncertainty estimation is calculated from Equations (7) and (8). This estimation takes into account all the probable experimental errors. Table 11 shows all uncertainty in the in-house calibration and sample measurement. Our data point to a combined standard uncertainty of 0.0098 with an expanded uncertainty of 2%. The expanded uncertainty has been used as a real confidence limit for each measurement for the proposed method.

## 6. Conclusions

A sensitive and accurate analytical method for validation of total-Hg in a wide range of environmental matrices was validated according to the IUPAC and Eurachem Guidelines.

The method developed displayed a broad dynamic range and low detection limit (>0.1 µg/kg) without the need for extensive sample preparation. The HotBlock digestion protocol utilised minor amounts of HNO_3_, H_2_SO_4_, and HF in a closed Teflon tube, thus reducing analyte loss and also reducing the risk of contamination. The preparation process was followed by CV AAS analysis. The obtained results were accurate and precise (Recovery > 90%, RSD ≤ 5%) when compared to the US-EPA method 3052 and 7474 when employed in the same samples.

## Figures and Tables

**Table 1:** The mean of eight replicate results of two different matrices (biota and marine sediment certified reference materials (CRMs)).

CRM	*n*	NIST-2976	Ref. Vale	PACS-3	Ref. Vale
Mean value (mg/Kg)	8	0.058 ± 0.002	0.061 ± 0.004	2.87± 0.15	2.98 ± 0.36

**Table 2:** Zeta results of two different matrices CRM.

CRM(NIST-2976)		CRM(PACS-3)	
Bias = −0.002		Bias = −0.28	
Relative bias = −4%		Relative bias = −9%	
*SD* = 0.006		u(lab) = 0.12	
u(ref) = 0.002		u(ref) = 0.25	
|z|	0.4	|z|	1.0

*SD*: Standard deviation.

**Table 3:** Comparison between our proposed method and traditional methods for total mercury analysis using cold vapour atomic absorption spectrometry (CV AAS) and comparable matrices.

Method	Matrix	Certified Value (µg/kg)	Avg. Measured Value (µg/kg)	Avg. % Rec	No. of Samples	RSD
EPA-7474	NIST 8406 SED	60	62	103%	70	15%
	NIST 1566 Oyster tissue	84	81	97%	72	15%
EPA-7473	Estuarine Sediment NIST SRM 1646	63	75	119%	N/A	3%
	Oyster Tissue NIST SRM 1566a	64	68	106%	N/A	3%

**Table 4:** Repeatability results in µg/L of five replicates at three different levels during one day.

	Level 1 (0.2 µg/L)	Level 2 (2 µg/L)	Level 3 (8 µg/L)
Mean	0.165	2.001	8.027
*SD*	0.008	0.030	0.077
RSD	5%	2%	1%

**Table 5:** Intermediate precision concentration in mg/kg of eight CRM replicates between 15 March and 17 April.

CRM	Mean/mg/kg	St. Dev.	RSD
PACS3	2.784	0.143	5.10%
NIST2976	0.062	0.003	4.80%

RSD: Relative standard deviation.

**Table 6:** Lack-of-fit test calculations for seven calibration points five times.

Slope	Intercept	*n*	*p*	Nominator	Denominator	*MSS_error_*	*MSS_lof_*
0.0316	0.0011	7	5	5	28	1.0 × 10^−5^	2.5 × 10^−6^

**Table 7:** Ruggedness studies of total mercury analyses in marine sediment samples (PACS3) according to Plackett–Burman test.

**Factor**	**Low Value (−1)**	**High Value (+1)**	**Experiment No.**	**Positive Effect**	**Negative Effect**	**Total Effect**	**Total Effect %**	**Mean Measured Values (mg/kg)**	**Ref. Value (mg/kg)**	**Recovery %**
Temp/°C	100	125	E1	3.07	−2.88	0.2	7%			
Digestion time/h	3	12	E2	3.17	−2.78	0.39	13%			
Dilution volume/mL	50	100	E3	3.17	−2.78	0.39	13%			
Sample wt./g	0.1	0.2	E4	2.69	−3.26	−0.57	−19%			
Reagents (HNO_3_ + H_2_SO_4_) mL	3 + 5	6 + 10	E5	2.85	−3.1	−0.26	−9%	2.97	2.98	99

**Table 8:** Ruggedness studies of total mercury analysis in biota samples (NIST2976) according to Plackett–Burman test.

**Factor**	**Low Value (−1)**	**High Value (+1)**	**Experiment No.**	**Positive Effect**	**Negative Effect**	**Total Effect**	**Total Effect %**	**Mean Measured Values (mg/kg)**	**Ref. Value (mg/kg)**	**Recovery %**
Temp/°C	100	125	E1	0.07	−0.09	−0.03	−0.90%			
Digestion time/h	3	12	E2	0.08	−0.08	−0.01	−0.30%			
Dilution volume/mL	50	100	E3	0.08	−0.08	0	0.10%			
Sample wt./g	0.1	0.2	E4	0.08	−0.08	0	−0.20%			
Reagents (HNO_3_ + H_2_SO_4_) mL	3 + 5	6 + 10	E5	0.07	−0.09	−0.01	−0.50%	0.065	0.061	107

**Table 9:** One-way analysis of variance (ANOVA) analysis results within biota CRM (NIST2976).

	Sum of Squares	df	Mean Square	*F*	Sig.
Between Groups	0.006	5	0.001	2.442	0.095
Within Groups	0.006	12	0.000		
Total	0.012	17			

**Table 10:** One-way ANOVA analysis results within sediment CRM (PACS-3).

	Sum of Squares	df	Mean Square	*F*	Sig.
Between Groups	0.370	5	0.074	2.213	0.121
Within Groups	0.401	12	0.033		
Total	0.771	17			

**Table 11:** Combined and relative expanded uncertainty calculations based on Type A and B evaluation.

Source of Uncertainty	Type	Measured Value	Error ±	Unit	*u_Relative_*	Probability Distribution	Divisor	Squared Standard Uncertainty (*u²*)
Repeatability of prepared 0.6 µg/L CRM	A	6 × 10^−4^	5.5 × 10^−6^	mg/L	9.2 × 10^−3^	Normal, 1s	1	8.5 × 10^−5^
Chemical Reagents purity	B	1.00	5.0 × 10^−3^		5.0 × 10^−3^	Rectangular	1.73	8.4 × 10^−6^
Pipette 1 mL repeatability	A	1.0 × 10^−3^	7.8 × 10^−8^	L	7.8 × 10^−5^	Normal, 1s	1	6.6 × 10^−9^
Temperature effect on Pipette 1 mL Volume	B	1.0 × 10^−3^	4.1 × 10^−7^	L	4.1 × 10^−4^	Rectangular	1.73	5.6 × 10^−8^
Calibration st certificate uncertainty of pipette 1 mL	B	1.0 × 10^−3^	3.4 × 10^−6^	L	3.4 × 10^−3^	Triangular	2.45	1.9 × 10^−6^
Measuring flask 1 mL repeatability	A	5.0 × 10^−2^	4.3 × 10^−6^	L	8.6 × 10^−5^	Normal, 1s	1	7.4 × 10^−9^
Temperature effect on flask 50 mL Volume	B	5.0 × 10^−2^	2.0 × 10^−5^	L	4.1 × 10^−4^	Rectangular	1.73	5.6 × 10^−8^
Uncertainty of flask 50 mL	B	5.0 × 10^−2^	6.0 × 10^−5^	L	1.2 × 10^−3^	Triangular	2.45	2.4 × 10^−7^
Concentration of the calibration standard	B	1001	2	mg/L	2.0 × 10^−3^	Normal, 2s	2	1.0 × 10^−6^
Tare weight repeatability	A	100	3.7 × 10^−3^	mg	3.7 × 10^−5^	Normal, 1s	1	1.3 × 10^−9^
Gross weight repeatability	A	100	3.7 × 10^−3^	mg	3.7 × 10^−5^	Normal, 1s	1	1.3 × 10^−9^
Balance linearity contribution (gross weight)	B	100	0.1	mg	1.0 × 10^−3^	Rectangular	1.73	3.3 × 10^−7^
Balance linearity contribution (tare weight)	B	100	0.1	mg	1.0 × 10^−3^	Rectangular	1.73	3.3 × 10^−7^
Balance readability (gross weight)	B	100	0.01	mg	1.0 × 10^−4^	Rectangular	1.73	3.3 × 10^−9^
Balance readability (tare weight)	B	100	0.01	mg	1.0 × 10^−4^	Rectangular	1.73	3.3 × 10^−9^
Combined standard *u_c_*	0.0098							
Expanded U at 95% confidence interval (*k* = 2)	2.0%							

**Table 12:** Fulfil acceptance criteria for all validation characteristics.

Parameter	Methodology	Acceptance Criteria	Results	Fulfil the Acceptance Criteria!
**Selectivity**	A specific and sharp peak of Hg produced during CRM analysis with no interferences	No interferences with the analyte peak.	Complies	Yes
**Trueness**	Relative bias was calculated for ten repeated CRMs	Relative bias shall not lie outside the limit of ±10%, and Zeta score shall less than or equal 2 to be satisfactory	Complies	Yes
**Recovery**	Two CRMs were used to calculate Recovery% = Measured value/Reference value.	±10% of the reference value	Complies	Yes
**Precision**	Repeated CRMs through one day of analysis and intermediate resection over a long time.	Relative standard deviation (RSD) shall not exceed 10%	Complies	Yes
**Limit of Detection and Limit of Quantitation**	Replicates of low-level concentration CRM matrix used to calculate SD (σ). Calculate *S*/*N* ratio	Limit of detection (3σ), limit of quantitation (10σ). *S*/*N* between 2.5 to 10	Complies	Yes
**Linearity:**	Independent calibration curves signal used to calculate (*F_Tab_*) and (*F_Cal_*).	Lack of fit test (Linear if *F_Cal_* < *F_Tab_*)	Complies	Yes
**Range**	The concentration interval over which linearity and accuracy are obtained and yields a precision of ≤3% RSD.	N/A	Complies	N/A
**Robustness**	Various conditions tested using Placket-Burman test for experimental design ruggedness. One-way ANOVA	One-way ANOVA was used as statistical acceptance criteria of robustness at 95% confidence interval	Complies	Yes
**Uncertainty of measurement**	Expanded Uncertainties were considered for in-house Calibration curve (*U_Cal_*) and sample measurement (*U_measu_*) to estimate the uncertainty from the main resources affecting the method.	U = k*u%	N/A	-
